# Understanding Addiction as a Developmental Disorder: An Argument for a Developmentally Informed Multilevel Approach

**DOI:** 10.1007/s40429-015-0079-2

**Published:** 2015-10-10

**Authors:** E. J. McCrory, L. Mayes

**Affiliations:** Developmental Risk and Resilience Unit, Division of Psychology and Languages Sciences, University College London, 26 Bedford Way, London, UK; Yale Child Study Center, 280 S. Frontage Road, New Haven, CT 06519 USA

**Keywords:** Childhood maltreatment, Addiction, Substance use disorders (SUDs), Epigenetics, Intervention

## Abstract

Substance abuse and drug addiction are two of the most common psychopathologies among the general population. While a host of risk factors are associated with the onset of drug abuse and drug addiction, there is a growing body of evidence pointing to the powerful influence of early adverse experiences, both child neglect and maltreatment, as well as drug use and abuse in parents and/or primary caretakers. We consider the case for drug addiction as a developmental disorder, outlining the need to consider the role of genetic, epigenetic, and neurobiological factors alongside experiences of adversity at key stages of development. Such a multilevel approach within a developmental framework has the potential to reframe our understanding of how addiction emerges and is maintained, and is essential if we are to identify the mechanisms underlying this disorder to better inform effective treatment and prevention across the generations.

Substance abuse and drug addiction are two of the most common psychopathologies among the general population. Across potential drugs of abuse, prevalence estimates in adult populations for drug abuse and dependence range from 1.4 % for 12-month to 7.7 % for lifetime drug abuse [1]. When the behavioral addictions are also considered (e.g., gambling, overeating), there is significant overlap in natural history, comorbidity, response to treatment, and etiologic mechanisms with drug use and abuse [2]. Many addictions begin in adolescence, which appears to be an especially vulnerable time for the onset of drug use and abuse and the transition to addiction [3]. There are also robust associations between the age of onset of drug use and abuse and the severity and chronicity of addiction [4]. While there are a host of risk factors associated with the onset of drug abuse and drug addiction, there is a growing body of evidence pointing to the influence of early adverse experiences, both child neglect and maltreatment, as well as drug use and abuse in parents and/or primary caretakers. Taken together, each of these lines of evidence suggest that drug addiction (and perhaps addictions more generally) may be construed as developmental disorders, that is, as disorders with experiential and gene by experience antecedents relating to early caregiving and exposure to adverse and/or contexts characterized by deprivation.

In delineating any developmental pathway to addiction, it is important to recognize the potential mechanisms by which such a pathway may begin as early as conception. While many women abstain from substance use during pregnancy, a significant number continue to use substances during this time and into the postpartum period. For those who do abstain, relapse rates are high in the initial months following delivery [5]. Further, substance abuse and addiction during pregnancy are commonly associated with chronic prenatal stress in mothers resulting in changes to maternal stress and immune systems that may have direct effects on similar systems in the fetus [6]. A growing body of human research indicates the deleterious effects of prenatal stress on birth outcomes and postnatal development [7] especially in the domains of emotion regulation that may predispose to greater risk for drug abuse and addiction. Thus, parental substance use appears to exert an influence on the following: (i) fetal development, which may be reflected in increased rates of preterm delivery, low birth weight, and multiple congenital abnormalities [8]; (ii) the prenatal environment construed more broadly as maternal health and stress management; (iii) the postpartum environment in which the infant is often in the continued care of a substance-abusing parent; and (iv) the adversity associated with high rates of neglect and abuse which has been reported in substance using parents [9]. High rates of abuse and neglect in turn expose children to chronic stress and adversity that has been linked to increased risk for later psychopathology including drug use.

Especially in reference to the postnatal caregiving environment, recent advances in neurobiological models of parenting report the re-wiring of key neural circuits of stress and reward by addiction that appear critical to supporting parenting [10•, 11•]. Indeed, for addicted parents, caring for a child is less rewarding and more stressful owing to the dysregulation between stress and reward neural circuits. This approach provides neurobiological data to accompany observational studies that have indicated decreased engagement and increased passivity between substance-using mothers and their newborn infants [12]. Thus, prenatal and postnatal parental substance use may have a detrimental effect on the developing child early on during infancy through several pathways but especially including those relating to how parental care impacts child stress regulatory capacities at the neural, psychobiological, immunologic, genetic, and endocrine levels and how those early perturbations in parental care are transmitted forward to the caring behaviors of those offspring when they later become parents themselves.

Recent data also suggest a prominent role for genetic as well as psychosocial factors in the transmission of substance abuse from parent to child [13, 14]. Moreover, there has been significant progress in identifying specific genes that influence substance abuse disorders. The search for specific genes has been aided by knowledge of developmental pathways leading to drug abuse. For example, the central importance of externalizing disorders in adolescence as a developmental pathway associated with higher rates of substance use and abuse has led to noting the importance of genes regulating the dopamine systems for both externalizing and alcohol disorders [15]. The newer techniques of genome-wide association studies have also underscored the importance of developmental process. Of special interest has been the identification of genes important in the earliest strategies of brain development which are associated with substance abuse much later on [16]. Moreover, genetic studies have been among the best documentation of the role of social factors in protecting or accelerating drug use. For example, the effect of genetic factors and delinquent behavior and alcohol use in boys are blunted in rural communities [17]. Recent comprehensive epidemiological studies also focus on the importance of the child’s experience of parental marriage: divorce serves as a major risk factor for subsequent alcohol abuse and dependence controlling for family history of substance abuse disorders [18].

The identification of endophenotypes such as impulsivity, which are risk factors for both externalizing and substance abuse disorders, has aided in gene identification [19]. For instance, a novel adoption design suggests that the first expression of a genetic risk for addictions and related impulsive behavior in infants and children may be problems in attention and behavioral self-control that can be ameliorated by parents with good emotional self-regulation who can provide appropriate structure [20, 21]. This is consistent with data suggesting that genetic vulnerability to substance use has been considered through the lens of self-control and emotion regulation [22]. In particular, impulsivity has been considered a key construct in the emergence of substance dependence during adolescence [23], with increasing levels of impulsivity and risky behavior more generally being higher during this developmental period. Notably, adolescence is marked by higher rates of experimental drug use, and substance use disorders begin to emerge [3, 24], an observation underscoring the importance of this period in the pathway to addiction before individuals even enter adulthood. Parents play a critical role in the socialization and regulation of emotions and behaviors in children, and children also shape and contribute to their parent’s own behavior regulatory functioning (e.g., [25, 26]). Indeed, families have their own capacities for self-regulation that may provide a protective effect against the transmission of substance abuse across generations [27]. Further, programs that focus on early parent-child relationships and closely related social processes have shown efficacy in preventing substance abuse (e.g. [28, 29]).

In sum, a growing body of research suggests a continuous set of circumstances from the intrauterine periods though infancy, childhood, and early adolescence through which individuals follow increasingly clear developmental pathways towards serious addictive disorders. The papers in this special issue each contribute distinct lines of evidence addressing the reframing of addictions as developmental disorders, focussing specifically on the following: (a) the role of gene-environment interactions in the emergence of specific addictive disorders; (b) the role of epigenetic mechanisms; (c) the role of early adversity in changing brain systems relevant to an addictive process; (d) the role of emotion regulation difficulties in key developmental periods such as adolescence in addiction and other psychopathologies; and (e) the use of developmentally and mechanism-informed intervention/prevention programs to reduce the risk for drug use and abuse across the developmental life span.

Milaniak, Watson, and Jaffee examine candidate gene studies that test gene × environment interactions with a focus on variants consistently associated with substance use and abuse. They specifically review the genes associated with nicotine, cannabis, and alcohol use and abuse with special attention to the genes linked to neural systems involved in addiction. What emerges is a mixed though provocative set of findings from quantitative behavioral genetic studies regarding gene × environment interactions in candidate gene studies. Importantly, while small, underpowered studies are surely problematic, the authors point to the challenge of obtaining adequately fine-grained measures of environmental factors in larger samples. Further, they underscore the need to guide genetic studies by biologically plausible mechanisms for drug use and abuse and for a more mechanistically informed understanding of environmental variables (e.g., more proximal measures of parental care behavior, the caring environment, or the impact of peer to peer interactions). Getting closer to developmental mechanisms also requires more translational research bringing together human and animal studies.

Cecil, Walton, and Viding address the role of epigenetic mechanisms in the onset of substance use disorders with a particular focus on DNA methylation. Across both human and animal studies, evidence generally supports an association with DNA methylation and substance use/addiction with the suggestion that developmental timing is key. The authors point to the limited knowledge today on the normal patterns in the methylome especially in humans and the variation by tissue, cell-type, gender, and age as well as the relative contribution of genetic and environmental influences on observed methylation patterns and a paucity of longitudinal studies beginning early with repeated assessments of methylation. That said, the authors report promising lines of evidence indicating that prenatal alcohol exposure is associated with increased methylation and decreased expression of a gene implicated in stress response, metabolism, and immune function. Such a finding provides one putative mechanism through which fetal programming can occur with prenatal alcohol exposure contributing to HPA axis dysregulation and increased addiction risk in adolescence and adulthood. While providing some preliminary clues regarding potential developmental mechanisms, epigenetic studies of addictive disorders are in their infancy and Cecil and colleagues make a compelling argument for a much more systematic study of the relationship between DNA methylation and addiction across tissues, substances, and developmental periods. The need for more longitudinal designs within human studies is clearly essential if we are to move from isolated findings regarding the role of DNAm in the pathophysiology of addiction to using such data as disease biomarkers that may inform therapeutic targets.

Puetz and McCrory examine the impact of early child maltreatment on key neural systems implicated in addiction mechanisms, namely reward processing, decision-making, and affect regulation. Early childhood adversity is associated with increased risk for a range of comorbid psychiatric disorders including substance use and addiction, and a growing body of work has demonstrated that childhood maltreatment is associated with functional and structural changes in these same systems. Adults presenting with addictive disorders show changes in these systems including heightened striatal response to salient stimuli and dampened fronto-cingulate regulation. Puetz and McCrory draw parallels with the remarkably similar pattern of findings in maltreated children and suggest that these early neurodevelopmental changes may in part account for the especially severe and chronic addiction profiles in adults with early histories of maltreatment. Specifically, they argue that early changes in brain structure and function following exposure to childhood maltreatment may instantiate increased latent vulnerability to addictive behavior in adolescence and adulthood [30•]. Alterations in neural systems involved in reward processing, decision-making, and affect regulation may have a proximal adaptive value early in development, but alterations may also incur long-term costs, increasing the risk for later psychopathology, including addiction.

With a perspective complementary to Puetz and McCrory, Shadur and Lejuez further explore developmental mechanisms by examining the relationship between emotional regulation in adolescence and risk for substance use and addiction. They highlight the need for understanding risk factors that cut across diagnoses and conditions, which may contribute to risk embedded across development. They cite *emotion regulation deficits* as a core transdiagnostic risk factor underlying the development of substance use, addiction, and comorbid psychopathology in adolescence. The dual-systems model of neurological development highlights adolescence as a critical period for increased risk for emotion regulation difficulties. In this model, the cognitive control system including prefrontal and parietal regions and the anterior cingulate is crucial to decision-making but is functionally dominated by a second affective system that includes regions which are important to processing reward and social and emotional salience, including but not limited to the amygdala, ventral striatum, orbitofrontal cortex, medial prefrontal cortex, and the superior temporal sulcus. The dominance of the affective system contributes to heightened reward sensitivity and a reward-seeking behavior, a crucial factor biasing decision-making and contributing to increased experimentation with drugs and the emergence of substance use/abuse. Shadur and Lejuez use two established developmental theories linked to emerging substance use disorders, the externalizing pathway and the internalizing pathway, which together highlight how early embedded risk in the form of emotion regulation deficits accounts for the development of addiction and comorbid psychiatric disorders. They also point to the implications for intervention, especially with adolescents, arguing for the particular need of addressing contextual stressors in higher risk developmental periods.

Finally, Fisher and Berkman continue the theme of mechanism-informed interventions by focusing on the transgenerational effects on the development of addictive disorders through interventions directly addressing the effects of early adversity. They make the argument that prevention efforts informed by the science of early adversity and chronic stress may reduce the population-level incidence of addictions. They propose an innovative framework for how translational neuroscience that is focused on the effects of early adversity may inform addictions intervention, describing an application of their proposed model in relation to smoking cessation. They argue that because of the common neural pathways of addiction, similar approaches to the one they illustrate for smoking cessation may prove applicable to other drug as well as behavioral addictions.

Taken together, these five papers begin to provide a compelling though incomplete case for addiction as a developmental disorder; however, much more longitudinal research is required with greater transdisciplinary and translational emphasis. Nevertheless, each of these papers offers a perspective on a developmental research agenda for a potentially more fruitful approach to understanding the mechanisms of addictions and their effective treatment and prevention across two and three generations.
